# Overexpression of Mutant Galactose Permease (*ScGal2*_N376F) Effective for Utilization of Glucose/Xylose or Glucose/ Galactose Mixture by Engineered *Kluyveromyces marxianus*

**DOI:** 10.4014/jmb.2008.08035

**Published:** 2020-10-08

**Authors:** Deok-Ho Kwon, Saet-Byeol Kim, Jae-Bum Park,, Suk-Jin Ha

**Affiliations:** 1 Department of Bioengineering and Technology, Kangwon National University, Chuncheon 2434, Republic of Korea; 2 Institute of Fermentation and Brewing, Kangwon National University, Chuncheon 4341, Republic of Korea; 3 Interdisciplinary Program in Biohealth-Machinery Convergence Engineering, Kangwon National University, Chuncheon 2441, Republic of Korea

**Keywords:** Cellulosic biomass, marine biomass, *Kluyveromyces marxianus*, xylose-specific transporter, ScGal2_N376F

## Abstract

Mutant sugar transporter *ScGAL2*-N376F was overexpressed in *Kluyveromyces marxianus* for efficient utilization of xylose, which is one of the main components of cellulosic biomass. *K. marxianus* ScGal2_N376F, the *ScGAL2*-N376F-overexpressing strain, exhibited 47.04 g/l of xylose consumption and 26.55 g/l of xylitol production, as compared to the parental strain (24.68 g/l and 7.03 g/l, respectively) when xylose was used as the sole carbon source. When a mixture of glucose and xylose was used as the carbon source, xylose consumption and xylitol production rates were improved by 195% and 360%, respectively, by *K. marxianus* ScGal2_N376F. Moreover, the glucose consumption rate was improved by 27% as compared to that in the parental strain. Overexpression of both wild-type *ScGAL2* and mutant *ScGAL2*-N376F showed 48% and 52% enhanced sugar consumption and ethanol production rates, respectively, when a mixture of glucose and galactose was used as the carbon source, which is the main component of marine biomass. As shown in this study, *ScGAL2*-N376F overexpression can be applied for the efficient production of biofuels or biochemicals from cellulosic or marine biomass.

## Introduction

As an alternative feedstock to fossil fuels, cellulosic biomass has been the focus of recent research because of its various advantages over edible biomass, *i.e.*, abundance and relatively cheap price [1-4]. However, several processes are required to use cellulosic biomass, and various substrates such as glucose and xylose must be produced. Therefore, it is necessary to be able to shorten these processes, and the employed strain should be capable of using various substrates. The strain *Kluyveromyces marxianus* is currently known to exhibit thermotolerance, making it an industrially robust yeast, because economical production processes, such as simultaneous saccharification and fermentation (SSF) or simultaneous saccharification and co-fermentation (SSCF), prefer high temperatures [5-7]. Above all, *K. marxianus* can utilize various substrates including xylose [6,8-11].

When coexisting with glucose as a substrate, xylose is not utilized substantially due to glucose catabolite repression [7,12-14]. The main reason for this problem is that xylose is competitively transported into the cell via some hexose transporters, which have higher affinity for glucose than xylose, at high concentrations of glucose [7, 14, 15]. In previous studies, the metabolic analysis of strains that were engineered for enhanced xylose fermentation in a glucose-xylose mixed-sugar substrate, revealed that the fermentation process is affected by not only the changes of xylose transport kinetics but also by increased levels of hexose transporter expression [16-19].

Studies on the *GXF1, SUT1, AT5G59250 (HP59), HXT7*, and *GAL2* genes of hexose transporters show potential for xylose utilization [15, 20, 21]. In particular, studies on *HXT7* and *GAL2* gene mutations using genetic engineering suggest many possibilities for the application of co-fermentation of glucose and xylose [15, 20, 21]. These results mean that one of the rate-limiting steps in xylose metabolism is xylose transport [16]. Consequently, the xylose-specific transporters, not being inhibited by glucose, are a crucial prerequisite for efficient and economical fermentation of xylose in the presence of glucose.

The mutant transporter ScGal2_N376F, derived from *S. cerevisiae*, has the highest preference for xylose [15, 22, 23]. In this study, the xylose-specific transporter gene, *ScGAL2*_N376F, was allowed to overexpress in the *K. marxianus* KCTC 17555*ΔURA3* strain, and the fermentation capabilities of the parental strain, *ScGAL2*-overexpressing strain, and *ScGAL2*_N376F-overexpressing strain were verified using only xylose, galactose, glucose, or a combination of these sugars as carbon source.

## Materials and Methods

### Strains and Plasmids

*S. cerevisiae* CEN.PK was used for cloning of galactose permease gene (*ScGAL2*). *Escherichia coli* TOP10 (KCTC 22006) was used for *ScGAL2* gene cloning and sequencing. *K. marxianus* KCTC 17555*ΔURA3* was kindly provided from Seoul National University. An overexpression plasmid, pJSKM316-GPD, was kindly provided from Sungkyunkwan University [24]. *K. marxianus* 17694-DH1 was obtained through a directed evolutionary approach and random mutagenesis in a previous paper [25].

### Cloning of *ScGAL2* Gene and Site-Directed Mutagenesis

The *ScGAL2* gene was amplified from genomic DNA of *S. cerevisiae* CEN.PK using *ScGAL2*_Fw and *ScGAL2*_Rv primers (Table 1). This amplified gene was used for T vector cloning, using a TOPcloner TA Core Kit (Enzynomics Inc., Korea) and then transformed into *E. coli* TOP10. Site-directed mutagenesis was performed for substitution of asparagine (N) at position 376 to phenylalanine (F) using a QuikChange Lightning Site-Directed Mutagenesis Kit (Agilent Inc., USA). The primers (*ScGAL2*_N376F_Fw and *ScGAL2*_N376F_Rv) used for the site-directed mutagenesis were designed using primer design software available online (Table 1).

### Construction of Expression Cassettes

Two pJSKM316-GPD vectors were constructed for expression of *ScGAL2* and *ScGAL2*_N376F in a *K. marxianus* strain. The target DNAs were amplified using *ScGAL2*_*XbaI*_Fw and *ScGAL2*_*XmaI*_Rv primers with a restriction enzyme site of *XbaI* and *XmaI* (Table 1). The PCR products and pJSKM316-GPD vector were digested with the enzymes, and both were ligated. The expression vector consists of *ScURA3* selection marker, GPD promoter, and TYC terminator.

### Transformation of *ScGAL2* Genes into *K. marxianus* KCTC 17555*ΔURA3* or *K. marxianus* 17694-DH1

Both *ScGAL2* and *ScGAL2*_N376F expression cassettes were amplified using primers (Table 1) ranging from *ScURA3* gene to CYC terminator. Each cassette was transformed into *K. marxianus* KCTC 17555*ΔURA3* or *K. marxianus* 17694-DH1 using an EZ-Yeast Transformation Kit (MP Inc., USA). *K. marxianus* transformants were selected in synthetic complete (SC drop-out plate including 6.7 g/l yeast nitrogen base w/o amino acids) (BD Difco Inc., France), 0.6 g/l CSM-His-Leu-Trp-Ura (MP Inc.), and 20 g/l glucose at 30°C.

### Fermentation Conditions

Pre-culture was performed in 5 ml of YP (10 g/l Yeast extract, 20 g/l Peptone) media including 20 g/l glucose (YPD_20_) at 30°C and 200 rpm. The cells were inoculated into 50 ml of YP media including 80 g/l glucose, xylose, or galactose (YPD_80_, YPX_80_, or YPGal_80_) at 30°C and 100 rpm. High-temperature fermentations were performed in YPX_80_ at 40°C and 100 rpm. Co-fermentation experiments were carried out in YP media including 40 g/l glucose and 40 g/l xylose (YPD_40_X_40_), 40 g/l glucose and 40 g/l galactose (YPD_40_Gal_40_) at 30°C and 100 rpm.

### Analytical Methods

Cell densities were measured at 600 nm using a GENESYS™ 10S UV-visible spectrophotometer (Thermo Inc., USA). Cells were centrifuged and the supernatants were analyzed by using a high-performance liquid chromatography (HPLC) system (Agilent Inc.) with a Rezex ROA-Organic Acid H^+^ column (Phenomenex Inc., USA) to measure the concentrations of glucose, xylose, galactose, xylitol, acetate, and ethanol. The temperatures of the column and refractive index detector (RID) were maintained at 50°C and 0.005 N of H_2_SO_4_ solution was used as a mobile phase at a flow rate of 0.6 ml/min.

## Results and Discussion

### Site-Directed Mutagenesis to Engineer a Xylose-Specific Transporter, ScGal2_N376F

Previously, it was reported that ScGal2_N376F mutant had the highest affinity for xylose, along with weakened affinity for glucose, among different hexose transporter mutants from engineered *S. cerevisiae* [15, 22]. *Ogataea polymorpha* yeast also exhibited improved xylose utilization during high-temperature alcoholic fermentation by overexpression of engineered *ScGAL2* mutants [23]. In this study, *ScGAL2* gene was cloned from *S. cerevisiae* and then *ScGAL2*_N376F gene was engineered by site-directed mutagenesis. Two adenines at positions 1126 and 1127 of *ScGAL2* gene were substituted with thymine, leading to amino acid substitution from asparagine (N) to phenylalanine (F) at position 376. Each expression cassette harboring *ScGAL2* or *ScGAL2*_N376F gene with GPD promoter, TYC terminator, and *ScURA3* gene as a selection marker, was introduced into *K. marxianus* 17555*ΔURA3* to confirm whether wild-type and mutant Gal2 possess different fermentation phenotype.

### Improved Xylose Fermentation Capability of Overexpressed *ScGAL2*_N376F Strain

**Fig. 1:**
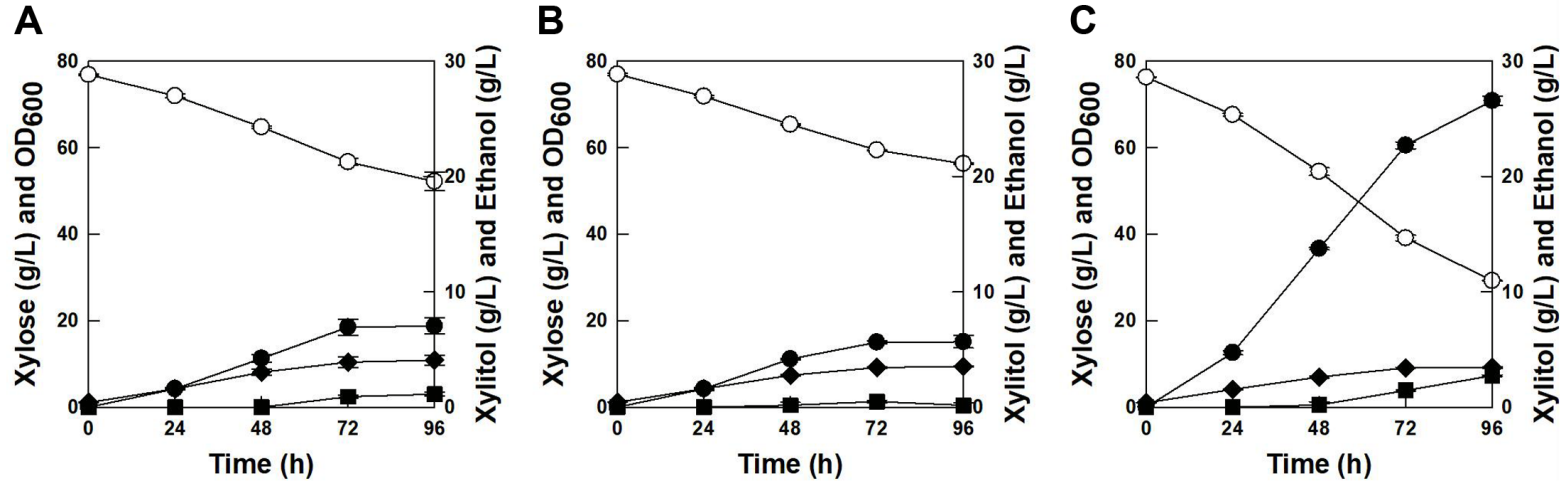
Time profiles of xylose fermentations by the parental strain (A), *K. marxianus* ScGal2 (B), and *K. marxianus* ScGal2_N376F (C) at 30°C and 100 rpm. Symbols: xylose (○), OD (◆), xylitol (●), and ethanol (■).

Fermentation experiments were performed to compare xylose fermentation capabilities between the parental strain (*K. marxianus* 17555*ΔURA3*) and engineered strains (*K. marxianus* ScGal2 or *K. marxianus* ScGal2_N376F) using xylose as a sole carbon source in YPX_80_ media. For fair comparisons between *K. marxianus* KCTC 17555*ΔURA3* and *K. marxianus* KCTC 17555*ΔURA3*+*ScURA3*, all fermentation results were nearly the same (data not shown). The parental strain consumed 24.68 g/l xylose and produced 7.03 g/l xylitol with a yield of 0.28 g/g for 96 h (Fig. 1A). The xylose consumption and xylitol production rates were 0.26 g/l/h and 0.07 g/l/h, respectively. Likewise, *ScGAL2* gene-overexpressing strain, *K. marxianus* ScGal2, exhibited similar xylose fermentation capability with the parental strain as shown in Fig. 1B. Xylose was consumed at 20.67 g/l for 96 h with a 0.22 g/l/h xylose consumption rate and 5.66 g/l xylitol was produced for 96 h by *K. marxianus* ScGal2. The xylitol yield and productivity of *K. marxianus* ScGal2 (0.27 g/g and 0.06 g/l/h) were almost similar to those from the parental strain. Interestingly, *ScGAL2*-N376F gene- overexpressing strain, *K. marxianus* ScGal2_N376F, showed highly enhanced xylose fermentation capability as shown in Fig. 1C. Xylose was consumed at 47.04 g/l, and 26.55 g/l xylitol was produced by *K. marxianus* ScGal2_N376F with the yield of 0.56 g/g. The xylose consumption rate and xylitol production rate were 0.49 g/l/h and 0.28 g/l/h, which were 91% and 278% improved, respectively, compared with those from the parental strain. In addition, the xylitol yield improved from 0.28 g/g to 0.56 g/g, which was a 2-fold improvement by *K. marxianus* ScGal2_N376F. These results suggest that substitution of asparagine (N) with phenylalanine (F) at position 376 of ScGal2 appreciably influences the xylose fermentation capability. Since XylE, xylose/H^+^ symporter of *E. coli*, showed good accordance with ScGal2 at transmembrane helices 5 (T219) and 8 (N376), changes at position 376 of ScGal2 could result in alteration of the binding pocket, and the N376F mutation would drastically reduce the space in the central cavity, which may offer an explanation for the xylose specificity of this mutant [15].

### Effects of *ScGAL2* Gene Overexpression on Galactose or Glucose Utilization

**Fig. 2:**
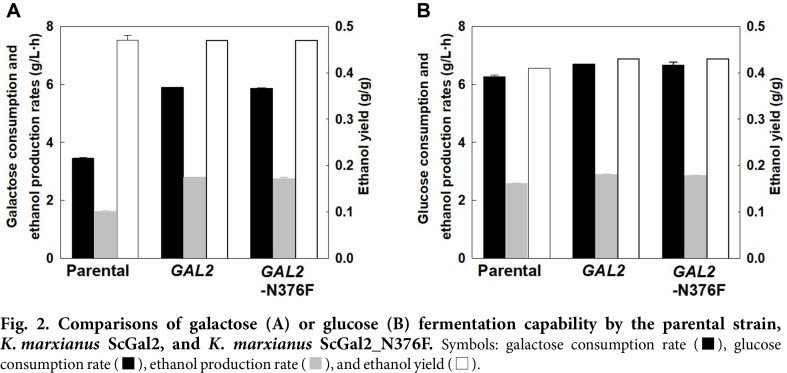


The parental strain and engineered strains fermented galactose or glucose as a sole carbon source in YPGal_80_ or YPD_80_ media, respectively, to verify the effect of *ScGAL2* gene overexpression on galactose or glucose utilization. When galactose was used as a sole carbon source, the parental strain consumed 80 g/l galactose within 24 h with a 3.45 g/l/h galactose consumption rate and a 1.62 g/l/h ethanol production rate (Fig. 2A). The two engineered strains *K. marxianus* ScGal2 and *K. marxianus* ScGal2_N376F exhibited much higher galactose consumption rates (5.90 g/l/h and 5.86 g/l/h) and ethanol production rates (2.79 g/l/h and 2.73 g/l/h), respectively, than those from the parental strain, because the two engineered strains consumed 80 g/l galactose much faster (within 14 h). Therefore, galactose consumption rates and ethanol production rates were 69~71% and 68~72% improved, respectively, by *ScGAL2* or *ScGAL2*-N376F gene overexpression. However, the ethanol yields from galactose were similar to the 0.47 g/g of the parental strain and the two engineered strains. These results indicated that *ScGAL2* or *ScGAL2*-N376F gene overexpression enhanced the overall galactose consumption rate to a comparable level without a change in ethanol yield.

When glucose was used as a sole carbon source, glucose consumption rates (6.69 and 6.66 g/l/h), ethanol production rates (2.88 and 2.85 g/l/h), and ethanol yields (0.43 and 0.43 g/g) were all slightly improved, respectively, by the two engineered strains as compared to those from the parental strain (6.27 g/l/h, 2.59 g/l/h, 0.41 g/g) as shown in Fig. 2B. These results indicated that overexpression of *ScGAL2* or *ScGAL2*-N376F gene, even slightly, improved the glucose consumption rate and ethanol yield by the two engineered strains.

The overexpression of the mutant *ScGAL2*-N376F gene resulted in improved xylose consumption rate, xylitol production rate, and xylitol yield as compared to those from the wild-type *ScGAL2* gene overexpression. However, the sugar consumption rate and ethanol production rate were not highly changed when galactose or glucose was used as a sole carbon source, suggesting that the N376F mutation of ScGal2 might not change the hexose sugar (glucose or galactose)-transporting capability, but might improve the pentose sugar (xylose)-transporting capability of the two engineered *K. marxianus* strains.

### Enhanced Mixed Sugar Utilization by *ScGAL2*_N376F-Overexpressing Strain

As yeasts display strong glucose catabolite repression when glucose and other sugars are used together, sequential utilization of a sugar mixture is a common phenomenon. Therefore, the rate of second sugar utilization highly affects overall productivity after glucose depletion. In this study, fermentation experiments using a sugar mixture (glucose/xylose or glucose/galactose) were performed to verify the effect of *ScGAL2* or *ScGAL2*-N376F overexpression for mixed-sugar fermentation.

**Fig. 3:**
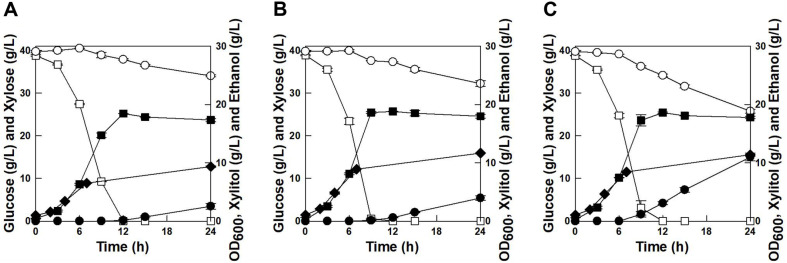
Comparisons of fermentation results in glucose-xylose mixture by the parental strain (A), *K. marxianus* ScGal2 (B), and *K. marxianus* ScGal2_N376F (C). Symbols: glucose (□), xylose (○), xylitol (●), OD (◆), and ethanol (■).

When a mixture of 40 g/l glucose and 40 g/l xylose was used as carbon source, the parental strain exhibited typical sequential utilization of the sugar mixture, which is characterized by fast consumption of glucose at first and very slow consumption of xylose later. *K. marxianus* ScGal2 and *K. marxianus* ScGal2_N376F also exhibited sequential utilization of glucose and xylose; however, the rates of utilization for each sugar were different. As shown in Fig. 3, the parental strain consumed 40 g/l glucose for 10 h and then slowly consumed 5.37 g/l xylose with 2.51 g/l of xylitol production for 24 h. *K. marxianus* ScGal2 exhibited almost similar fermentation results as the parental strain, however, only glucose consumption rate was higher than that in the parental strain. *K. marxianus* ScGal2_N376F exhibited higher glucose and xylose consumption rates than those in the parental strain. Through the overexpression of *ScGAL2*-N376F into *K. marxianus* strain, the overall consumption rate of the glucose-xylose mixture, ethanol productivity, and xylitol productivity were improved by 22%, 3%, and 340%, respectively. These results suggest that the overexpression of *ScGAL2*-N376F in the yeast strain could be very suitable for the utilization of cellulosic biomass composed mainly of glucose and xylose. Since the parental strain (*K. marxianus* 17555*ΔURA3*) possesses an inefficient xylose metabolic pathway, only *K. marxianus* ScGal2_N376F showed improved xylose consumption rate and xylitol production rate. If an efficient xylose-fermenting yeast was used as the parental strain, enhanced ethanol production rate might be achieved along with enhanced xylose consumption rate.

When a mixture of 40 g/l glucose and 40 g/l galactose was used as carbon source, *K. marxianus* ScGal2 and *K. marxianus* ScGal2_N376F also exhibited faster glucose and galactose utilization rates that those from the parental strain as shown in Table 2. The parental strain consumed 40 g/l glucose for 10 h and then consumed 40 g/l galactose for 20 h with 35.39 g/l ethanol production. Both *K. marxianus* ScGal2 and *K. marxianus* ScGal2_N376F consumed 40 g/l glucose for 8 h and then consumed 40 g/l galactose for 14 h with 36.14~36.63 g/l ethanol production. The overexpression of the wild-type *ScGAL2* or the mutant-type *ScGAL2*_N376F into *K. marxianus* increased both glucose and galactose consumption rates in a glucose-galactose mixture. Therefore, the overall consumption rate of the glucose-galactose mixture, ethanol productivity and ethanol yield, were improved by 48%, 52%, and 2.9%, respectively, by the overexpression of *ScGAL2*-N376F into *K. marxianus* strain. These results suggest that *K. marxianus* ScGal2_N376F could be a very suitable yeast strain for the utilization of marine biomass composed mainly of glucose and galactose.

### Xylose fermentation capability was highly improved by overexpression of *ScGAL2*-N376F into *K. marxianus* 17694-DH1

**Fig. 4:**
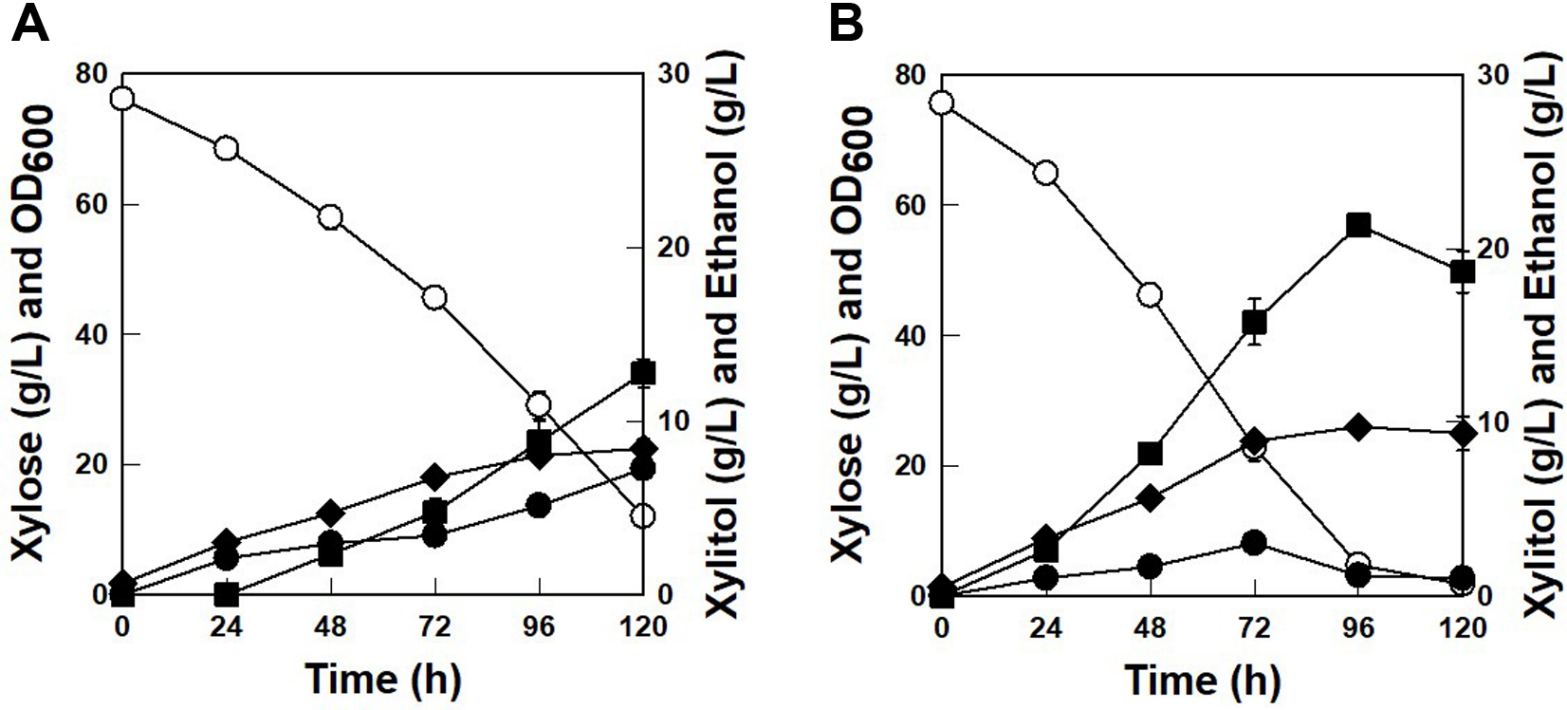
Time profiles of xylose fermentations by the parental strain (A) and *K. marxianus* 17694-DHG1 (B) at 30°C and 200 rpm. Symbols: xylose (○), OD (◆), xylitol (●), and ethanol (■).

According to Fig. 3, the effect of *ScGAL2*-N376F gene overexpression for ethanol production from xylose is not clear because the parental strain (*K. marxianus* KCTC 17555*ΔURA3*) is not an efficient xylose-fermenting strain. Therefore, *ScGAL2*-N376F gene was overexpressed into *K. marxianus* 17694-DH1 which showed more efficient ethanol production capability from xylose, to verify the effect of *ScGAL2*-N376F overexpression on xylose fermentation [25]. The *ScGAL2*-N376F-overexpressed strain, *K. marxianus* 17694-DHG1, exhibited a 0.49 g/l/h xylose consumption rate and a 0.09 g/l/h ethanol production rate, representing 151% and 242% improved results, respectively, as compared to those from the parental strain, *K. marxianus* 17694-DH1 (Fig. 4). In addition, ethanol yield was improved from 0.18 g/g to 0.30 g/g by *K. marxianus* 17694-DHG1. When *ScGAL2*_N376F was overexpressed with several key enzymes for xylose utilization into *K. marxianus*, the engineered strain showed co-consumption of glucose and xylose [7, 26]. However, only *ScGAL2*-N376F-overexpressed strain *K. marxianus* 17694-DHG1 did not show co-consumption of glucose and xylose which suggested that the overexpression of only *ScGAL2*-N376F into *K. marxianus* is not enough for the co-consumption of glucose and xylose.

## Conclusion

The efficient utilization of sugar mixtures such as glucose/xylose or glucose/galactose is a prerequisite for the production of fuels and chemicals from cellulosic or marine biomass by engineered yeast. When the mutant sugar transporter *ScGAL2*-N376F was overexpressed in *K. marxianus*, xylose utilization rate and xylitol production rate were improved by 195% and 360%, respectively, with glucose consumption rate improved by 27% as compared to that in the parental strain, when a glucose/xylose mixture was used as a carbon source. *K. marxianus* ScGal2_N376F also exhibited a 48% improved overall consumption rate of glucose/galactose mixture and 52%improved ethanol productivity as compared to that in the parental strain. These results suggest that the *ScGAL2*-N376F-overexpressing strain could be very suitable to produce fuels and chemicals from cellulosic or marine biomass.

## Figures and Tables

**Table 1:** Primers used in this study.

Primer names	Sequence (5'→3')
*ScGAL2*_Fw	ATGGCAGTTGAGGAGAACAATATGCC
*ScGAL2*_Rv	TTATTCTAGCATGGCCTTGTACCACG
*ScGAL2*-N376F_Fw	TCCATTGTCATTGGTGTAGTCTTCTTTGCCTCCACTTTCTTTAG
*ScGAL2*-N376F_Rv	CTAAAGAAAGTGGAGGCAAAGAAGACTACACCAATGACAATGGA
*ScGAL2*_*XbaI*_Fw	GCTCTAGAATGGCAGTTGAGGAGAACAATATGCC
*ScGAL2*_*XmaI* _Rv	TCCCCCCGGGTTATTCTAGCATGGCCTTGTACCACG
Fw_scURA3	CGG CAT CAG AGC AGA TTG TAC TGA GAG TGC
Rv_CYCt	CCT CAC TAA AGG GAA CAA AAG

**Table 2:** Comparisons of fermentation results by engineered *K. marxianus* strains in YP medium containing glucose-galactose mixture.

Results of fermentation	Parental strain	*K. marxianus* ScGal2	*K. marxianus* ScGal2_N376F
OD	11.80 ± 0.07	19.63 ± 0.25	18.98 ± 0.60
Ethanol	35.39 ± 0.00	36.63 ± 0.26	36.14 ± 0.82
Glucose consumption rate	4.00 ± 0.10	5.00 ± 0.00	5.00 ± 0.00
Galactose consumption rate	0.98 ± 0.06	2.86 ± 0.00	2.82 ± 0.00
Ethanol production rate	1.69 ± 0.02	2.62 ± 0.02	2.58 ± 0.06
Ethanol yield	0.44 ± 0.00	0.46 ± 0.00	0.45 ± 0.01
